# Transcriptional deregulation of genetic biomarkers in *Chironomus riparius* larvae exposed to ecologically relevant concentrations of di(2-ethylhexyl) phthalate (DEHP)

**DOI:** 10.1371/journal.pone.0171719

**Published:** 2017-02-06

**Authors:** Óscar Herrero, Gloria Morcillo, Rosario Planelló

**Affiliations:** Grupo de Biología y Toxicología Ambiental, Facultad de Ciencias, Universidad Nacional de Educación a Distancia, UNED, Paseo de la Senda del Rey 9, Madrid, Spain; Hokkaido Daigaku, JAPAN

## Abstract

Di(2-ethylhexyl) phthalate (DEHP) is a ubiquitous environmental pollutant used worldwide as a plasticizer and solvent in many formulations. Based on available toxicological data, it has been classified as toxic for reproduction and as an endocrine disruptor. Despite this, ecotoxicological studies in aquatic wildlife organisms are still scarce. In the present work, the toxic molecular alterations caused by DEHP in aquatic larvae of the midge *Chironomus riparius* have been studied, by analyzing the transcriptional activity of genes related to some vital cellular pathways, such as the ribosomal machinery (*rpL4*, *rpL13*), the cell stress response (*hsc70*, *hsp70*, *hsp40*, *hsp27*), the ecdysone hormone pathway (*EcR*), the energy metabolism (*GAPDH*), and detoxication processes (*CYP4G*). Environmentally relevant concentrations (10^−3^ to 10^5^ μg/L) and exposure conditions (24 to 96 h) have been tested, as well as the toxic effects after DEHP withdrawal. Although the compound caused no mortality, significant changes were detected in almost all the studied biomarkers: e.g. strong repression of *hsp70*; general inhibition of *EcR*; *GAPDH* activity loss in long exposures; among others. Our data show a general transcriptional downregulation that could be associated with an adaptive response to cell damage. Besides, the activity of the compound as an ecdysone antagonist and its delayed effects over almost all the biomarkers analyzed are described as novel toxic targets in insects.

## Introduction

Phthalates or phthalic acid esters (PAEs) are a family of man-made chemicals widely used since 1930s as plasticizers in the manufacture and processing of plastic materials, and which also appear in the composition of hundreds of consumer products, such as paints, detergents, adhesives, solvents, lubricants, insecticides, cosmetics and personal care products, among others [1,2]. World production of PAEs has grown rapidly in recent decades and, to date, 6–8 million tons of these chemicals are consumed worldwide each year [3]. Among all of them, di(2-ethylhexyl) phthalate (DEHP) has been the most commonly used, with an approximate annual consumption at present of 1–2 million tons [4,5].

Given that PAEs are not chemically bound to the polymeric matrix, they can gradually enter the environment by losses during their manufacturing, storage, use, and disposal [6,7], and can leach, migrate or evaporate into indoor air and atmosphere, soil, water, and a variety of materials such as foodstuff or medical devices, among others [1,8–10]. These characteristics, along with their physico-chemical properties, have turned PAEs into ubiquitous environmental pollutants that represent serious risks to human and environmental health.

Based on its toxicological profile and along with other phthalates, DEHP is blacklisted as a priority substance by the European Union [11,12], and several international regulatory agencies [3,8,13]. This chemical has also been included in different regulations concerning water quality, in which agencies have established the highest concentrations in water that are not expected to involve a significant risk to the majority of species in a specific environment, or to people [12,14,15]. Moreover, the presence of DEHP or other PAEs in different consumer products (e.g. toys, foodstuff) has been banned or restricted in recent years [16–18], and the presence of DEHP in drinking water has been severely limited [19–21].

Because of the large variety of toxic effects described to date, DEHP is classified as toxic for reproduction [22,23], Endocrine Disrupting Chemical (EDC) [24], and possibly carcinogenic to humans [5]. The environmental fate and toxicokinetics of DEHP has been reviewed extensively [1,3,8,25]. In its main degradation pathway, DEHP hydrolyzes to mono-ethylhexyl phthalate (MEHP) and follows subsequent glucuronid conjugation, but the formation rate and fate of MEHP in the environment is not known [26]. Differences in detoxication capabilities among aquatic species lead to variable bioaccumulation rates, although it is known that invertebrates are less able to break down DEHP [27]. MEHP reprotoxic effects have been reported in studies on mammals, but there are no other data on ecotoxicological properties of MEHP available [26]. Despite the increase in recent years in the amount of data available on the environmental toxicity of DEHP, research on its ecotoxicological effects in aquatic invertebrates and benthic wildlife organisms is still limited.

Since water constitutes the main vehicle for the dispersion of DEHP, aquatic ecosystems are especially sensitive to the presence of the compound. Although the compound in water tends to bind to sediments and suspended particles, a small amount persists dissolved in the water column [27]. Sediments integrate time-space effects of surface water pollution and represent a serious hazard to benthic and pelagic communities, so the ability of the compound to accumulate in such material makes it essential the evaluation of its toxic effects on species in that specific environment [26]. In this regard, among all freshwater benthic invertebrates, chironomid larvae constitute one of the most ubiquitous and abundant, and represent an important link in the food chain, capable of incorporating pollutants accumulated in sediments in which they grow and feed on. In fact, *Chironomus* midges are internationally used as model organisms in standardized environmental toxicity protocols [28–33], and have been selected as suitable organisms for research on the capacity of xenobiotics to cause endocrine disruption [34].

Complementary to other scientific works with more classical toxicity endpoints (e.g. survival, growth, immobilization, life-cycle, etc.), the use of molecular targets for ecotoxicity assessments (e.g. gene or enzyme activity) has increase in recent years in *Chironomus riparius*, as they have demonstrated to be effective biomarkers for the early detection of cellular stress responses and chemical toxicity, and constitute an important approach to achieve time and cost-effective tests for larger-scale evaluations. As an example, in the last few years different *Chironomus* species have served to assess transcriptional alterations caused by exposure to phthalates and other xenobiotics, biocides, metals, and nanoparticles, among other environmental stressors (e.g. [35–40] and references therein). It has been described the modulation of genes encoding ribosomal proteins, heat-shock proteins, hemoglobins, the ecdysone receptor, the estrogen-related receptor, alcohol dehydrogenase, calponin, and serine-type endopeptidase (e.g. [41–45] and references therein). Although it has been shown that expression levels of some of these molecular biomarkers are physiologically modulated throughout the development [42], available data demonstrate that gene expression is more sensitive to toxicant exposure than life cycle endpoints, underlining that the transcriptional changes can be harnessed to diagnose the exposure and effects of environmental chemicals in ecotoxicity testing and environmental risk assessments [46–48].

To determine accurate responsive genes which could be used as reliable biomarkers in the ecotoxicological risk assessment of DEHP, our objective in the present study was to analyze its molecular effects on aquatic larvae of the model organism *C*. *riparius*. We selected environmentally relevant concentrations and different exposure scenarios and evaluated the transcriptional activity of genes related to several crucial cell systems: the ribosomal machinery (*rpL4*, *rpL13*); the cell stress response (*hsc70*, *hsp70*, *hsp40*, *hsp27*); the ecdysone hormone pathway (*EcR*); the energy metabolism (*GAPDH*); and detoxication processes (*CYP4G*). We also assessed the enzyme activity of glutathione S-transferase (GST).

## Materials and methods

### Test animals

The experimental animals were aquatic larvae from the non-biting midge *Chironomus riparius*. Larvae used were reared under standard laboratory conditions, according to toxicity testing guidelines [32,33,49]. They were grown from egg masses in polyethylene tanks (500 mL) with aqueous culture medium (0.5 mM CaCl_2_, 1 mM NaCl, 1 mM MgSO_4_, 0.1 mM NaHCO_3_, 0.025 mM KH_2_PO_4_, 0.01 mM FeCl_3_) supplemented with nettle leaves, commercial fish food, and cellulose tissue. Cultures were maintained under constant aeration at 20°C and standard light-dark periods 16:8. Experiments were carried out using exclusively fourth instar larvae, and the larval stage was determined based on the size of head capsule [49].

### Exposure conditions and survival tests

Solutions of di(2-ethylhexyl) phthalate (DEHP, CAS No. 117-81-7) (Sigma-Aldrich, USA) were dissolved in analytical grade ethanol to provide a stock concentration of 10^7^ μg/L. The test solutions were constructed in culture medium at 0.01% ethanol. This was the final percentage of ethanol present in the solvent controls used in the experiment and preliminary tests demonstrated that induced no effects on the organisms in any of the selected endpoints. The nominal concentrations of DEHP ranged from 10^−3^ to 10^5^ μg/L, and included both higher and lower levels than those described in the literature for drinking water resources and aquatic ecosystems [1,3,8,19,20,26].

Larval survival was studied in all DEHP concentrations for 24-h exposures, and at the four lowest doses (10^−3^ to 1 μg/L) for longer experiments. The delayed toxicity (24+24 h), consisting of 24-h exposure followed by DEHP withdrawal and additional 24 hours in fresh medium, was also evaluated. Groups of 20 larvae were selected randomly and exposed to aqueous solutions of DEHP, without sediment. Larvae were not fed during the experiments, and survival rates were calculated after 24, 48, 72, or 96 h. For each experimental condition, four independent experiments were performed and groups of five surviving larvae were randomly selected, stored at -80°C, and used for RNA or protein extraction.

### RNA and protein isolation

TRIzol Reagent (Invitrogen, Germany) was used to extract total RNA from frozen larvae, following the manufacturer's instructions. Samples were treated with RNase-free DNase (Roche, Germany) for 90 min, and an organic extraction with phenol-chloroform was completed. Following precipitation using isopropyl alcohol (0.5 v/v) and washing with 70% ethanol, RNA was resuspended in DEPC water, quantified by absorption spectroscopy (BioPhotometer, Eppendorf, Germany), and stored at -80°C.

Total protein content was obtained after homogenization in 0.5 mL of Tris–EDTA buffer (40 mM Tris, 1 mM EDTA, pH 7.8) with 7x complete EDTA-Free protease inhibitor (Roche) with a subsequent centrifugation [42], and was quantified with BCA Protein Assay Reagent (Thermo Fisher Scientific, USA), according to the manufacturer's instructions. Protein samples were stored at -20°C.

### Reverse Transcription Polymerase Chain Reaction (RT- PCR)

Gene expression analyses were carried out only at low DEHP doses (10^−3^ to 1 μg/L). Semi-quantitative RT-PCR was used to evaluate the mRNA expression profile of selected genes. Reverse transcription was performed with 1 μg of the isolated RNA. Oligo(dT) primer (Invitrogen) was used with the M-MLV enzyme (Invitrogen) following the manufacturer’s instructions. Suites of primers were selected to specifically target selected genes (Table 1). PCR was performed in a MiniOpticon Thermocycler (Bio-Rad, USA), according to [42]. For all samples, the initial cDNA content and the amplification curves were adjusted in the PCR protocols, to prevent the ulterior saturation of DNA bands and subsequent quantification errors. The amplified PCR products were run in a 9% acrylamide gel at 60 V for 3 hours in 1x TGE buffer (40 mM Tris-Cl (pH 8.5), 200 mM glycine, and 2.5 mM EDTA), visualized after ethidium bromide staining and quantified with Chemigenius3 (Syngene, USA), using GeneSnap 6.05 and GeneTools 3.06 software. Actin and 26S were used as reference genes to normalize the fluorescence of bands, and a second normalization was carried out to calculate the expression levels of DEHP-exposed samples in relation to control conditions. To minimize technical errors, three replicates were carried out for each experiment.

**Table 1:** Primers used for RT-PCR amplification of the genes studied in *C*. *riparius*. Forward (F) and reverse (R) sequences, base pair (bp) length of the obtained fragments and origin of sequences are provided. References to the original published sequences can be found in [38,42].

Gene	Description	DNA sequence (5′-3′)	Fragment size (bp)
*actin*	Actin protein	F GATGAAGATCCTCACCGAACG	201
		R CGGAAACGTTCATTACCG	
*26S*	26S ribosomal ribonucleic acid	F TTCGCGACCTCAACTCATGT	220
		R CCGCATTCAAGCTGGACTTA	
*rpL4*	Ribosomal protein L4	F AACGCTTCAGAGCTGGACGTGG	149
		R ATTCATCTTGTGTACGCTCATTG	
*rpL13*	Ribosomal protein L13	F AAGCTGCTTTCCCAAGAC	351
		R TTGGCATAATTGGTCCAG	
*hsc70*	70 kDa heat-shock cognate protein	F CGTGCTATGACTAAGGACAA	239
		R GCTTCATTGACCATACGTTC	
*hsp70*	70 kDa heat-shock protein	F CATGTGAACGAGCCAAGAGA	274
		R TTGCCACAGAAGAAATCTTG	
*hsp40*	40 kDa heat-shock protein	F TACGTGACGCTAGAGGAAA	131
		R TTCCAGCCCGGCTT	
*hsp27*	27 kDa heat-shock protein	F TCCTCGTGCTTGCC	202
		R CAAGGATGGCTTCCA	
*EcR*	Ecdysone receptor	F AGACGGTTATGAACAGCC	240
		R CGAGCCATGCGCAACATC	
*GAPDH*	Glyceraldehyde 3-phosphate dehydrogenase	F GGTATTTCATTGAATGATCACTTTG	110
		R TAATCCTTGGATTGCATGTACTTG	
*CYP4G*	Cytochrome p450 family 4 subfamily G	F GACATTGATGAGAATGATGTTGGTG	340
		R TAAGTGGAACTGGTGGGTACAT	

### Glutathione S-Transferase (GST) activity

Two representative DEHP concentrations were selected to evaluate the GST activity: 1 and 10^3^ μg/L. For each experiment, five control larvae and five treated larvae were collected after DEHP treatments. Three replicates were performed and each sample was run in duplicate wells. Total protein was quantified with BCA Protein Assay Reagent (Thermo Fisher Scientific), and 25 μg of total protein were used for the enzyme assay. The GST activity was assessed spectrophotometrically with the kit GST (Sigma-Aldrich) in a JASCO V-530 spectrophotometer (JASCO, Japan), following [42].

### Data analysis

SPSS® Statistics 22 software (IBM, USA) was used for statistical analysis. Normality (Shapiro-Wilk’s test) and homoscedasticity (Levene’s test) of data were checked. The normalized levels of transcripts and the GST enzyme function were analysed with ANOVA, followed by Games Howell’s or Bonferroni’s *post hoc* tests, when appropriate. The Kruskal-Wallis’ test was used when data were not homogeneous or normally distributed, and the differences between pairs were stablished using Mann-Whitney’s tests. Significant differences were stablished at p < 0.05.

## Results

### Larval survival

A wide range of concentrations (10^−3^ to 10^5^ μg/L) was selected to assess larval mortality after 24-h exposure to DEHP. Within this range, levels found in natural exposure scenarios and also exposure concentrations evaluated in laboratory studies were tested [2,9,26,27,50,51]. For longer treatments (48 to 96 h), the four lowest concentrations were selected. Larval survival was not affected under any of the conditions analyzed. Although in 24-h exposures no significant mortality was observed, reaching survival rates very close to 100%, all experiments in these conditions showed a generalized loss of mobility and coloring of individuals in concentrations above 1 μg/L. These effects were not observed in longer exposures to lower concentrations.

### Expression profile of ribosomal genes

**Fig 1:**
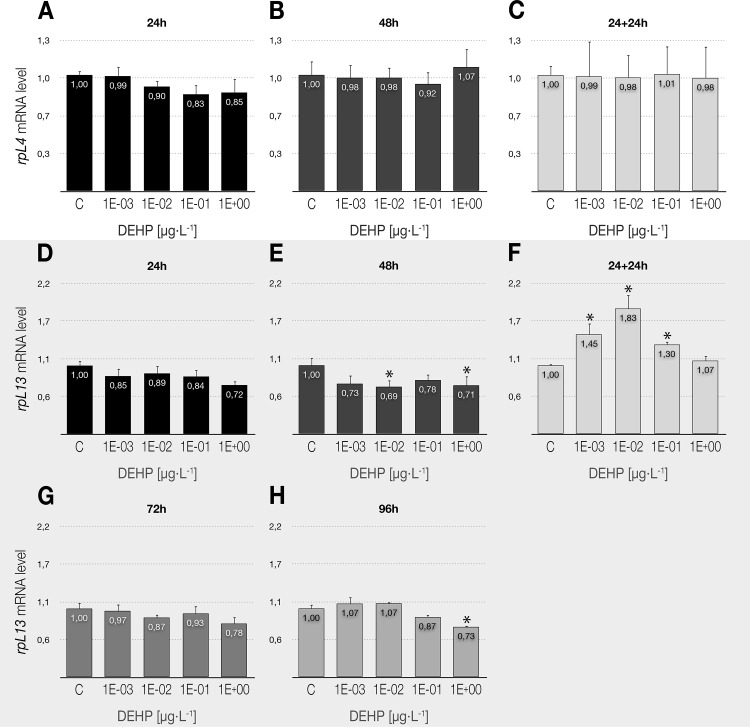
**Effects of DEHP treatments on the relative expression of genes coding for ribosomal proteins: *rpL4* (A-C), and *rpL13* (D-H).** Each bar is the mean ± SE obtained from three independent experiments, each with three replicates. Values are expressed as fold changes with respect to the control. X-axis values range from 10^−3^ to 1 μg/L. *Significant differences (p ≤ 0.05) as compared to control cultures.

The ability of DEHP to alter the ribosomal machinery was evaluated by means of transcriptional analysis of genes encoding ribosomal proteins L4 and L13. As shown in Fig 1A–1C, no effects were detected for the *rpL4* gene in none of the studied conditions. However, the *rpL13* gene was affected by the xenobiotic in all exposure scenarios, confirming the slight downregulation (not significant) detected in 24 and 72 h (Fig 1D and 1G) as statistically significant at 48 and 96 h (Fig 1E and 1H). Particularly relevant was the clear upregulation of *rpL13* in the delayed toxicity experiments (24-h exposure to DEHP plus 24 h in fresh culture medium) (Fig 1F), showing the ability of DEHP to stimulate the transcriptional activity of this gene time after removal from culture medium.

### Alteration of the heat-shock transcriptional response

**Fig 2:**
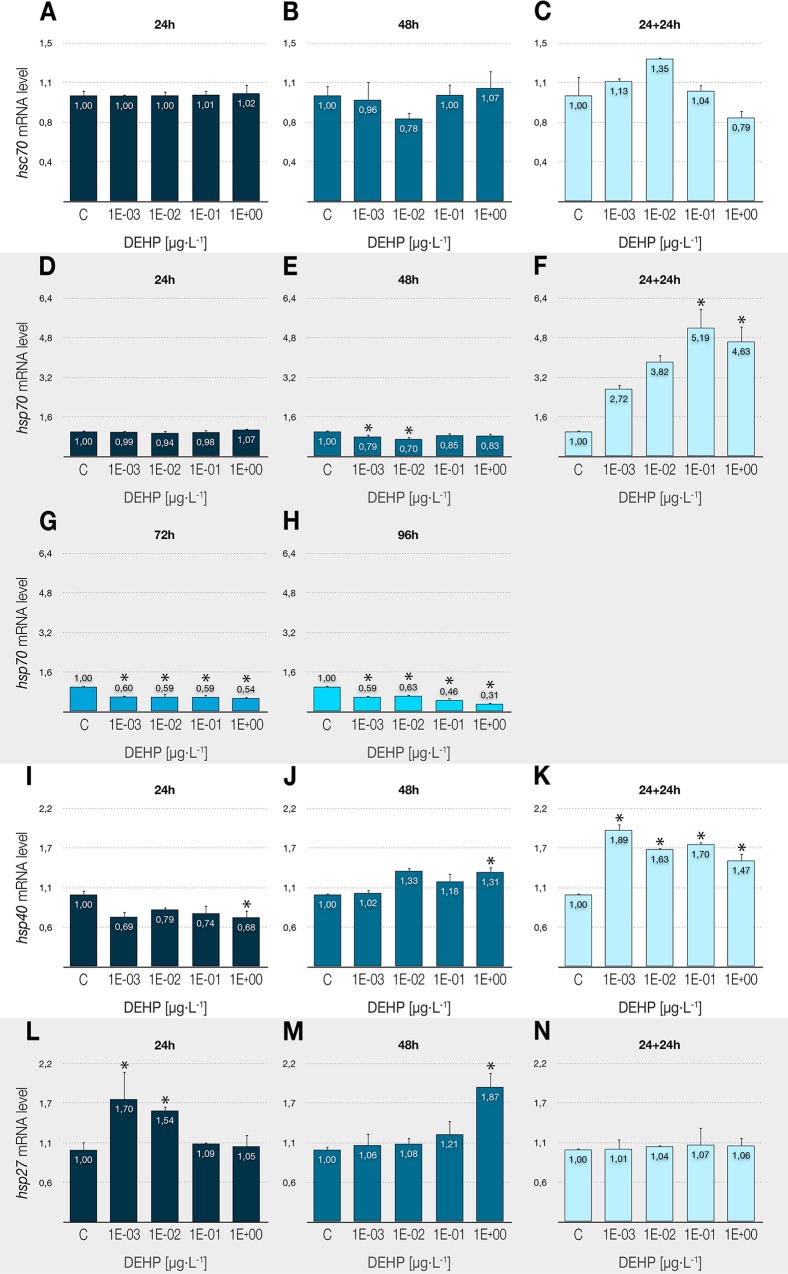
**Effects of DEHP treatments on the relative expression of different heat-shock genes in *C*. *riparius* fourth instar larvae: *hsc70* (A-C), *hsp70* (D-H), *hsp40* (I-K), and *hsp27* (L-N).** Each bar is the mean ± SE obtained from three independent experiments, each with three replicates. Values are expressed as fold changes with respect to the control. X-axis values range from 10^−3^ to 1 μg/L. *Significant differences (p ≤ 0.05) as compared to control cultures.

The expression profile of constitutive (*hsc70*) and inducible (*hsp70*, *hsp40*, and *hsp27*) heat-shock genes were analyzed to evaluate the interactions of DEHP on the cellular stress response. Although no significant changes were detected for the heat-shock cognate gene *hsc70*, as shown in Fig 2A–2C, significant transcriptional alterations caused by DEHP were found for all inducible heat-sock genes. The compound induced a clear dose- and time-dependent downregulation of the *hsp70* gene in exposures over 24 h, reaching values of 70% below control in 96-h treatments (Fig 2E, 2G and 2H). Earlier effects were detected in the genes coding for the 40 and 27 kDa proteins, with a repression of *hsp40* at the highest dose (Fig 2I) and the overexpression of *hsp27* at the lowest ones (Fig 2L). For these two genes, 48-h exposures led to increased transcriptional levels comparing with control samples (Fig 2J and 2M), unlike that observed for *hsp70*. Delayed toxicity tests revealed a strong overexpression of *hsp70* (up to 5-fold) (Fig 2F), and a slight but steady transcriptional induction of *hsp40* (Fig 2K).

### Modulation of the ecdysone receptor levels

**Fig 3:**
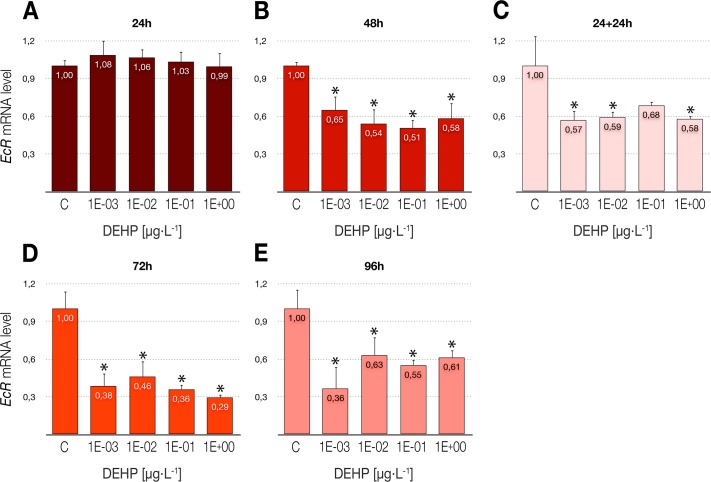
Effects of DEHP treatments on the relative expression of the ecdysone receptor gene (*EcR*) in *C*. *riparius* fourth instar larvae. Each bar is the mean ± SE obtained from three independent experiments, each with three replicates. Values are expressed as fold changes with respect to the control. X-axis values range from 10^−3^ to 1 μg/L. *Significant differences (p ≤ 0.05) as compared to control cultures.

The endocrine disrupting effect of DEHP and its ability to behave as a hormone antagonist was evaluated by means of the analysis of the transcriptional levels of the gene encoding the ecdysone receptor (*EcR*). Like the effects previously described for the *hsp70* gene, DEHP exposures led to a time-dependent downregulation of *EcR*, with significant reductions of the transcriptional activity in each exposure time (48 to 96 h) for all the studied concentrations (between 30% and 70% below control, respectively), as shown in Fig 3A, 3B, 3D and 3E. However, contrary to that observed for the *hsp70* gene, it is noteworthy that the withdrawal of the compound after 24-h treatments triggered a significant repression of the *EcR* gene 24 h later (Fig 3C), thus reaching transcriptional values about 40% below control in the delayed toxicity studies.

### Detoxication and energy metabolism

**Fig 4:**
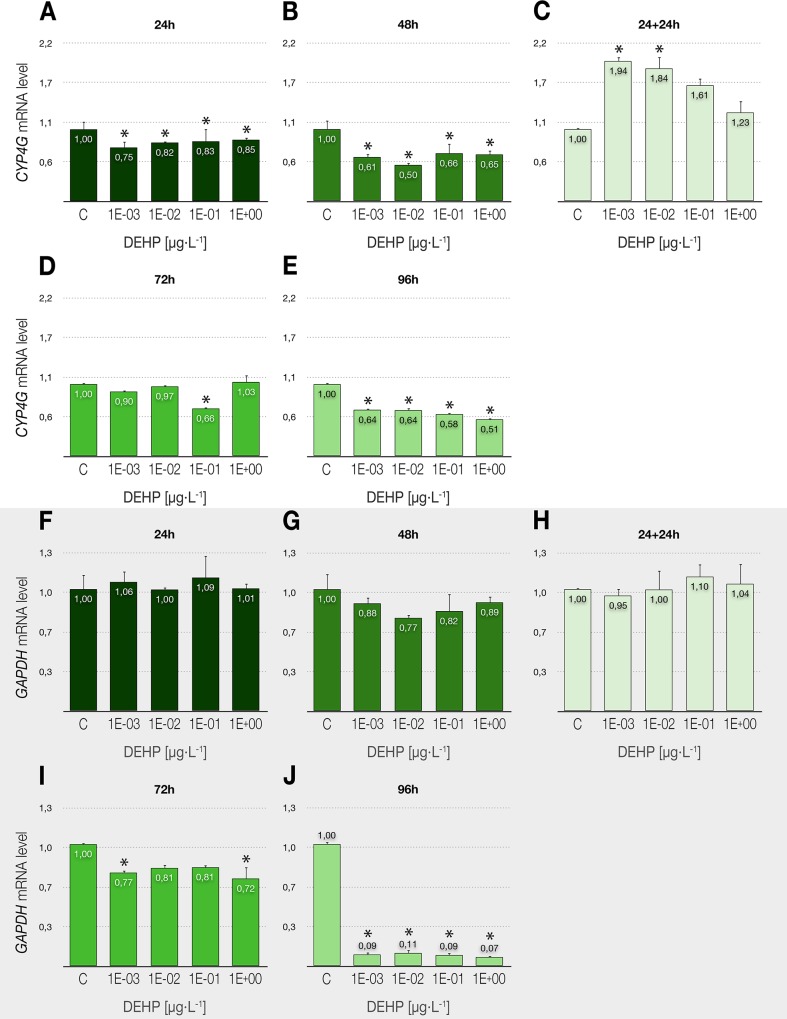
**Effects of DEHP treatments on the relative expression of genes involved in the detoxication metabolism in *C*. *riparius* fourth instar larvae: *CYP4G* (A-E), and *GAPDH* (F-J).** Each bar is the mean ± SE obtained from three independent experiments, each with three replicates. Values are expressed as fold changes with respect to the control. X-axis values range from 10^−3^ to 1 μg/L. *Significant differences (p ≤ 0.05) as compared to control cultures.

**Fig 5:**
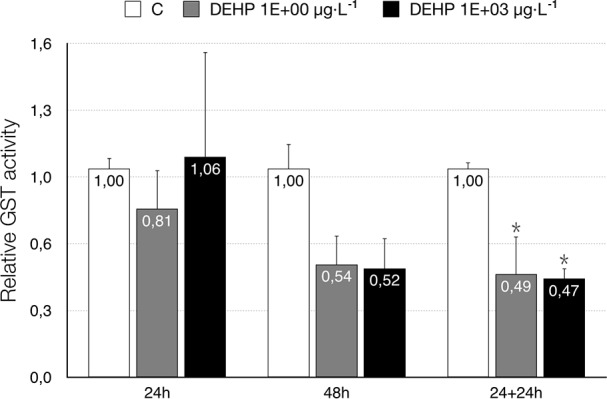
Glutathione S-transferase activity in *C*. *riparius* fourth instar larvae after exposure to DEHP (1 and 10^3^ μg/L) for 24, 48, and 24+24 hours. Each bar is the mean ± SE obtained from three independent experiments, each with three replicates. Values are expressed as fold changes with respect to the control. *Significant differences (p ≤ 0.05) as compared to control cultures.

The expression pattern of the *CYP4G* gene, as well as the enzyme activity of GST, provided us data related to the detoxication activities in *C*. *riparius*, whereas the transcriptional levels of *GAPDH* showed the possible toxic interaction between DEHP and the energy metabolism of exposed larvae. The xenobiotic clearly repressed the activity of CYP4G, even in the lowest concentrations at the shortest exposure time (24 h) (Fig 4A), and this decline remained significant for longer experiments (Fig 4B and 4D), with values close to 50% below control in 96-h treatments (Fig 4E). It is important to note that the repression produced by DEHP after 24 h was not only completely reverted after its removal but turned into a significant overexpression in the delayed toxicity assays (Fig 4C). The other detoxication pathway analyzed, represented by the GST enzyme (Fig 5), showed no effects in the 24-h acute exposures but was reduced after 48 h in the concentrations studied (1 and 10^3^ μg/L), with identical effects in both 48-h continuous exposure experiments and delayed toxicity tests.

Finally, DEHP had a time-dependent effect on the transcriptional activity of the *GAPDH* gene (Fig 4F–4J), leading to a significant reduction in 72-h treatments (Fig 4I) and an almost absolute repression in 96-h exposures (Fig 4J), with values of about 90% below the control. No delayed toxicity was detected for this gene (Fig 4H) in none of the studied conditions.

## Discussion

DEHP is by far the most commonly used plasticizer. Its high production volume and its many applications worldwide make this chemical a ubiquitous pollutant, allowing the emergence of numerous possible exposure scenarios. The scientific community has frequently discussed the ability of the compound to produce adverse effects in humans. Its toxic and carcinogenic effects have been well described in experimental animals (especially rodents) [5,26,50] and plants [52]; however, its ability to induce similar effects in humans is still controversial [53]. In contrast to the abundance of studies focused ultimately on human health, there is much less information in scientific literature concerning the ecotoxicological profile of DEHP, particularly in invertebrates.

MEHP is the primary biodegradation product of DEHP and has proven to be toxic in studies with mammals. It is therefore reasonable to believe that MEHP could cause toxic effects also to other species like birds, fish, frogs etc. [26] However, very scarce information is available and, particularly in invertebrates, there are no previous works assessing the toxic effects of this DEHP metabolite. In this sense, further research is needed to evaluate the consequences of MEHP exposure on invertebrate species.

### Survival analysis

Exposures to DEHP spanned from 24 to 96 hours and comprised a wide range of concentrations (10^−3^ to 10^5^ μg/L), although the five highest concentrations were tested only for short-term (24h) survival experiments. This conditions include DEHP levels both above and (especially) below those described in other scientific works concerning drinking water resources or aquatic environments [8,9,26,51]. Interestingly, the survival rate of *C*. *riparius* larvae was not significantly affected in any of the conditions tested, which contrasts with the significant mortality caused by butyl benzyl phthalate (BBP) in experiments performed under identical conditions, where the LC50 was established at 2.7·10^4^ μg/L [42]. These results are in accordance with the lack of significant mortalities detected for other invertebrate species (*Hyalella azteca*, *Chironomus tentans*, *Lumbriculus variegatus*) [54,55], even in long-term toxicity tests (*Dahpnia magna*, *Chironomus plumosus*, *Palamonetes pugio*, *Mytilus edulis*) [56–62].

Although no mortality was detected under any exposure condition, 24-h exposure to concentrations above 1 μg/L led to a generalized loss of mobility and coloring of individuals, proportionally to increased DEHP concentrations. Though not the subject of our study, larval mobility is considered a parameter equivalent to death rate in some ecotoxicological assays [63]. Similarly, larvae discoloration could be used as an early indicator of the toxicity of a compound [64], given that loss of red pigmentation, typically associated with hemoglobin, is a representative characteristic of larvae exposed to hypoxia-induced stress conditions [65]. The ability of DEHP to reduce the transcriptional activity of different hemoglobin genes has been previously described in *C*. *tentans* [44,45]. Although these genes were not included among the biomarkers selected for this work, the observed effects bring an interesting target for future studies regarding the toxicity of the xenobiotic.

### Transcriptional changes on the expression of ribosomal protein genes

Genes encoding ribosomal proteins have evidenced to be sensitive biomarkers in *C*. *riparius* larvae exposed to different stressors ([38] and references therein). The stability shown by both *rpL4* and *rpL13* in 24-h exposures, confirms that the ribosomal machinery does not seem to be an early target of the toxic effect of low doses of the xenobiotic, in accordance with the results described by [66] for the ribosomal genes *rpS3*, *rpS6*, *rpL11*, *rpL13*, and *rpL15* in a higher DEHP concentration. Our results demonstrate the capacity of DEHP to alter the activity of genes encoding ribosomal proteins by means of a slight but significant downregulation of *rpL13*, similarly to that observed in a previous work with the phthalate BBP [38]. It should be noted that removal of the compound resulted in a clear overexpression of the *rpL13* gene, which could indicate that the organism increases its ribosomal activity (protein synthesis) trying to compensate for the damage induced previously by the xenobiotic, as described previously under other stressful conditions (e.g. [67]).

### Alterations on the cellular stress response

The 70 kDa gene family includes both constitutive (*hsc70*) and inducible (*hsp70*) members, which share many common structural characteristics but present quite different expression patterns. While the constitutive forms are expressed at stable and relatively high levels under any condition, inducible heat-shock genes present a very low transcriptional profile under normal conditions, but their expression and subsequent translation of their products increase rapidly in response to a plethora of stress signals [68,69]. Our results showed no effects in *hsc70* or *hsp70* transcriptional levels after 24-h exposure to DEHP concentrations up to 1 μg/L. A previous work in *C*. *tentans* [45] found a clear induction of both genes after 24 h, at concentrations from 5·10^2^ μg/L. This was partially consistent with another research in *C*. *riparius* [41] where DEHP led to the upregulation of *hsp70* at concentrations between 10^3^ and 10^5^ μg/L, although *hsc70* remained unaltered. In our study, longer exposures to the xenobiotic induced time and dose-dependent changes in the transcriptional profile of *hsp70*, with a strong and significant downregulation for all DEHP concentrations at 72–96 hours (up to 70% below control). It has been described that downregulation of *hsp70* levels leads to increased sensitivity towards apoptosis-inducing agents, induces differentiation and cell death in cancer cells [70], and diminishes cell survival [71], although the strong repression detected in our study (together with those detected for *rpL13*, *EcR*, *CYP4G*, and *GAPDH*) could be the result of a general protective cell response consistent in maintaining a general low transcriptional profile in the presence of a sustained damage [72]. It is important to note the absence of changes in the activity of *hsc70* in our experimental conditions, which reinforces the idea of its constitutive presence and confirms the toxic role of DEHP in the specific transcriptional alterations observed for the other genes.

The HSP40 protein is important for protein folding/unfolding, translation, translocation, and degradation, as it stimulates the ATP-ase activity of the HSP70 proteins [73]. In addition, the HSP40/HSP70 chaperone complex controls specific processes at distinct locations within the cell (e.g. cell cycle, cell differentiation, or apoptosis), including the progression of certain pathologies (e.g. oncogenesis, viral infections) [70]. Thus, the slight repression of *hsp40* gene after 24-h exposure could compromise the effective course of HSP70 role, while in 48-h treatments the gene was slightly overexpressed, therefore promoting this chaperone activity. Our results clearly contrast with previous data [74] in which short-term exposures to three concentrations of DEHP (1, 10, and 30 μg/L) induced significant increases (up to 4-fold) in the expression of *hsp40* and *hsp90*.

The HSP27 protein belongs to the small molecular weight heat shock proteins (sHSPs) family, and plays two major roles in response to stressful stimuli: (1) preserves the regular functioning of cells through remodelling and stabilization of the cytoskeleton, as well as facilitating the proper refolding or removal of defective proteins; and (2) prevents apoptosis by interfering with caspase activation in both mitochondrial dependent and independent pathways, and also lowering the levels of reactive oxygen species [75]. The chaperone activity of HSP27 prevents the formation of aggregates of denatured or improperly folded proteins [76], which can serve as a pro-apoptotic signal [77]. Such misfolded forms are processed by the endoplasmic reticulum (ER), which is responsible for the structural maturation of proteins by means of two different mechanisms that try to avoid ER stress: the unfolded protein response (UPR), which increases the folding capacity; and the ER-associated degradation (ERAD), which leads to protein removal [78]. It has been recently described that DEHP can trigger the ER stress response [79], leading to an adaptive response (inhibition of cell proliferation, cell cycle delay) rather than a pro-apoptotic one [80]. Previous works in *C*. *riparius* have detected a variety of *hsp27* transcriptional responses under temperature or xenobiotic-induced stress ([42,81] and references therein). In the present study, the *hsp27* gene tended to a significant overexpression in treatments from 24 to 48 hours, that could counteract the apoptotic signals derived from *hsp70* downregulation.

Thereby, taken together, our results could suggest that DEHP is somehow altering protein folding and thus activating an adaptive response that slows down metabolism, trying to buy enough time to correct errors before progressing with normal cell cycle. Additionally, in vertebrates the HSPs are involved in stabilizing and activating the steroid hormone receptor [82], so if similar functions are performed in arthropods, changes in the transcriptional activity of these genes, particularly *hsp70*, could result in the alteration of the ecdysone-mediated hormone pathway by affecting the hormone receptor [83].

### Effects over the ecdysone hormone pathway

Phthalates have been identified as EDCs in humans and also in mammalian models [84], causing adverse effects on reproduction and development. Although it has been described that *EcR* transcriptional peaks occur naturally during larval development [85,86], different studies have demonstrated the ability of some xenobiotics to modulate the expression levels of the receptor, working as ecdysone agonists/antagonists and ultimately influencing the development of the organism (e.g. [42,87,88]). Moreover, although endocrine systems in invertebrates differ drastically from vertebrates, it is worth mentioning that ecdysteroid hormones in insects belong to the family of steroid hormones, and that the ecdysone receptor belongs to the superfamily of nuclear hormone receptors that includes estrogens, androgens, thyroid hormone, retinoic acid and glucocorticoid receptors, among others [41]. Consequently, the ability of the *EcR* gene to detect endocrine disrupting activities in invertebrates may be a useful tool in an attempt to prevent environmental risks derived from EDCs, and also in predicting possible interferences with the vertebrate hormone system. In a previous work in *C*. *riparius* [41], 24-h exposure to DEHP did not alter the transcriptional levels of *EcR* in concentrations up to 10^4^ μg/L, although a slight significant repression was detected at 10^5^ μg/L. Results obtained in the present work demonstrate that longer exposures (48 to 96 hours) also lead to a significant downregulation of this gene, although at concentrations that are up to eight orders of magnitude below than those reflected in that previous study. Therefore, our data suggest that DEHP interferes with the endocrine function acting as an ecdysone antagonist, both at short exposures to high doses, or at long exposures to low doses. Data obtained for 48-h exposures are similar to those obtained with BBP [42], which could indicate that under certain conditions this antagonistic effect could be common to several members of the phthalate family.

### Variations in energy and xenobiotic metabolism

Classic targets in ecotoxicity testing (e.g. survival, growth, reproduction rate) ultimately reflect changes in the energy metabolism of organisms [89]. Under this assumption, the transcriptional profile of the gene encoding GAPDH enzyme has been studied in the present work, as this protein plays a key role in energy production throughout glycolysis, although it is also involved in other functions at multiple subcellular compartments [90]. Precisely because of its many functions and its constant expression, *GAPDH* has been widely used as a reference gene in RT-PCR quantification, although it is known that it can be unstable under various experimental conditions (e.g. [91]). This fact makes necessary to check the stability of *GAPDH* under every experimental condition, thus avoiding errors caused by incorrect normalization. Our results show a stable behavior of the gene up to 48-h exposure to DEHP, but a significant drop in its transcriptional levels after 72 hours. It should be emphasized the almost total absence of *GAPDH* activity in 96-h experiments (up to 99% below control), which could be consistent with the hypothesis about the ability of the xenobiotic to slow down metabolism as an adaptive response to DEHP toxic effects.

In addition to their detoxication functions, P450 enzymes are also involved in the synthesis of ecdysteroids and juvenile hormones, with key roles in insect growth, development, and reproduction [92]. Thus, the response of some cytochromes seems to establish relations between exposure to some chemicals and the endocrine function [93]. Alterations in the transcriptional activity of different *CYP* genes have been described previously in *Chironomus* larvae exposed to a variety of xenobiotics ([42] and references therein). Our experiments let us detect a significant repression of the *CYP4G* gene in all the tested concentrations, even at the shortest DEHP exposures. This downregulation was concomitant with the significant low levels detected for *EcR* after 48 to 96-h exposure to DEHP, which could reflect the previously described association between the enzyme activity, the ecdysone synthesis and, by extension, the hormone receptor levels.

In many species, the expression levels of *GST* genes and the GST enzyme activity can be boosted significantly following exposure to xenobiotics, suggesting that they are involved in adaptive responses to chemical stress. Studies on the insect GSTs have focused primarily on their role in conferring resistance to insecticides [94]. As an example in chironomids, there have been described GST alterations in the gene activity [95] or the enzyme function [42,96,97] in response to the presence of xenobiotics. In this work, we have evaluated only two representative DEHP concentrations (1 and 10^3^ μg/L) and it was found that this phthalate inhibits GST enzyme activity to about 50% at both doses in 48-h experiments, similarly to that observed for *CYP4G* transcriptional rates. This could suggest that DEHP restricts the ability of *C*. *riparius* larvae to maintain normal detoxication rates, decreasing the biotransformation capacity of both phase I and phase II metabolism enzymes.

### Delayed toxicity studies

These experiments showed that the compound can cause adverse effects to the selected targets even after its withdrawal. Although some biomarkers that were not altered in 24-h exposures remained unchanged after 24 h of recovery (*rpL4*, *hsc70*, *GAPDH*), other genes showed a significant upregulation once the compound was removed from the culture medium (*rpL13*, *hsp70*, *CYP4G*). It could be hypothesized that the end of the DEHP injury allows the organism to recover normal metabolic rates, therefore being necessary to reinforce the ribosomal machinery and the protein folding/checking systems. Moreover, in the knowledge that high *hsp70* levels lead to increased resistance against apoptosis [70], its upregulation could be compensating this protective role, implemented during the presence of the xenobiotic by high levels of the *hsp27* gene. It is also possible that the significant rise in *hsp40* levels is directly related to the increased activity of the HSP40/HSP70 chaperone complex. It should be highlighted that the removal of DEHP revealed surprising findings about the endocrine disrupting capacity of this phthalate. Although the transcriptional activity of the *EcR* gene was not altered in 24-h exposures to DEHP, its removal led to a significant repression of this gene when larvae were maintained an additional 24 h in fresh culture medium. This behavior differs from the rest of genes analyzed in this work and appears to confirm a specific effect of the xenobiotic on the endocrine system of *C*. *riparius*, acting as an antagonist of the insect steroid hormone.

## Conclusions

The present study indicates that DEHP, the most commonly used plasticizer in the world, induces transcriptional alterations in exposed larvae of *C*. *riparius*. These toxic effects occur after short acute exposures, even at concentrations lower than those detected in environmental samples or permitted by different international regulations for drinking water. The use of biomarker genes constitutes a useful tool for the early detection of toxic effects, especially for those cellular pathways that have been evolutionary-conserved across different species. It is of particular interest to see how some of the toxic effects are detected long after the larvae have ceased to be exposed to the compound, which makes some of the DEHP toxic properties usually go unnoticed in the acute exposures of classical toxicity tests. Given the importance of this non-biting midge in the trophic chains of aquatic ecosystems, and the ability of the compound to bioaccumulate, it is advisable to continue investigating the different toxic effects of this xenobiotic, especially in natural populations. In addition, the appearance of effects at such low concentrations poses a potential worldwide risk to human and environmental health.

## Supporting information

S1 Fig Graphical abstract of the presented work. Acute exposures (24 to 96h) to a wide range of DEHP concentrations (1 ng/L to 0.1 g/L) caused no mortality in *C*. *riparius* larvae but led to a loss of mobility and coloring, and to a general decrease in the transcriptional activity of the studied genes.
(TIF)
