# Supplementary figures and images for: Transcriptional deregulation of genetic biomarkers in Chironomus riparius larvae exposed to ecologically relevant concentrations of di(2-ethylhexyl) phthalate (DEHP)
Source: PLoS One. 2017 Feb 6;12(2):e0171719. doi: 10.1371/journal.pone.0171719 (PMC5293269; doi:10.1371/journal.pone.0171719)

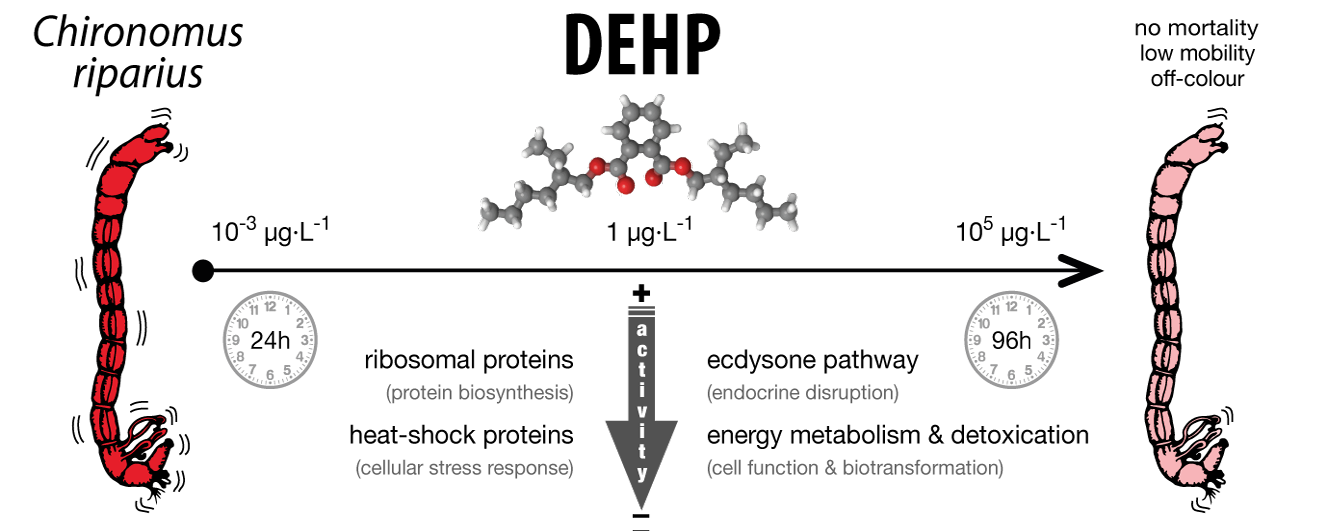

Supplement: S1 Fig — Acute exposures (24 to 96h) to a wide range of DEHP concentrations (1 ng/L to 0.1 g/L) caused no mortality in C. riparius larvae but led to a loss of mobility and coloring, and to a general decrease in the transcriptional activity of the studied genes. (TIF) [file pone.0171719.s001.tif]
